# Comparing Physical Intimacy and Romantic Relationships of Autistic and Non-autistic Adults: A Qualitative Analysis

**DOI:** 10.1007/s10803-023-06109-0

**Published:** 2023-08-16

**Authors:** Giorgia Sala, Jessica Hooley, Merrilyn Hooley, Mark A. Stokes

**Affiliations:** 1 Healthy Autistic Life Lab, School of Psychology, Deakin University, 1 Gheringhap St, Geelong, VIC 3220 Australia; 2 Dept Psychological Sciences, Swinburne University, John Street Hawthorn, VIC, Melbourne, 3122 Australia

Autism Spectrum Disorder (ASD) is a neurodevelopmental condition persisting through the lifespan, characterised by difficulties with social-emotional reciprocity across various contexts, and restricted or repetitive interests, behaviours or activities (American Psychiatric Association, 2013). According to surveillance studies in the United States, approximately 1 in 36 children is diagnosed with an ASD (Maenner et al., 2023). In this paper, identity-first language will be used as it is often preferred by many self-advocates (Kenny et al., 2016). The term “autistic” will be used to describe those diagnosed with an ASD (and potentially other comorbidities), and those without a diagnosis of ASD or other neurodiverse diagnoses will be referred to as “non-autistic” (N-A). The authors acknowledge that the terms “non-autistic” and “neurotypical” can be unclear as these groups may include people who have sub-clinical symptoms or undiagnosed mental/neurodevelopmental disorders.

Research into ASD and sexuality has grown significantly in recent years, and it has become widely acknowledged that autistic people have similar levels of interest in romantic relationships as non-autistic people (Hancock et al., 2020; Yew et al., 2021). Many autistic people have current or previous relationship experience (Byers et al., 2013; Strunz et al., 2017) which contrasts with historical perspectives wherein the difficulties of autistic people were seen to exclude them from having sexual and romantic relationships (e.g., Torisky & Torisky, 1985), and where research largely focused on proxy reports of whether sexual behaviour and interest existed in this population (Konstantareas & Lunksky, 1997; Ousley & Mesibov, 1991; Realmuto & Ruble, 1999). There is now a stronger focus on autistic peoples’ subjective understandings and experiences, and using these to identify ways to support themin navigating their personal and sexual identity to maximise positive outcomes (Dewinter et al., 2020). This collaborative approach to understanding sexuality and ASD has revealed increased rates of non-heterosexual attraction^1^, as well as gender variance and dysphoria among autistic people, which has obvious implications in requiring specific, tailored support (Genovese, 2021; George & Stokes, 2016; Sala et al., 2020b).

Given the growing acknowledgement of sexuality and relationship interest among autistic people, qualitative explorations of lived experience provide ways for researchers to gain a deeper understanding of how autistic people experience their own sexuality and relationships. For instance, Sala et al. (2020) explored enablers and barriers of emotional intimacy in romantic relationships among autistic (*n* = 31) and non-autistic (*n* = 26) people, who were recruited to respond to open-ended questions about their lived romantic experiences. Thematic analysis was utilised to identify key themes, similarities, and differences across the two groups. Several themes facilitating emotional or relational intimacy for both groups were identified. These ‘Enablers’ were captured as *Communication, Sharing and Similarity*, *Respect and Awareness: Self and Other*, and ‘*Work in Progress’*. Aspects of these themes were shared between autistic and non-autistic participants, but differences also emerged. For example, both groups shared a preference for open and honest discussion of issues, but autistic participants emphasised theneed for explicit and clear communication which was not an issue identified by non-autistic participants. Themes emerging as ‘Barriers’ to emotional intimacy included *Conflict: Intrapersonal and Interpersonal*, for both autistic and non-autistic participants, and *Uncertainty*, which was unique to the autistic group.

Other qualitative research has highlighted other important issues, such as perceived inadequacy in sexuality education, uncertainty about understanding their own and other’s sexual orientation (Hannah & Stagg, 2016), as well as wanting more “practical” sexuality and relationship education (Cheak-Zamora et al., 2019). Dewinter et al. (2017) identified positive aspects of sexual development in young autistic males, that involved exploration with others, seeking information through various channels, and increasing personal body-awareness. And Barnett and Maticka-Tyndale (2015) used semi-structured online interviews with 24 autistic adults to explore sexual experiences and sexual education. Using thematic analysis, they identified themes of difficulty with courtship and flirting, sensory processing differences that interacted with desire and/or ability to engage in sexual practices, and insufficient sexual education. Participants in their study outlined strategies used to overcome sensory issues to enjoy sexual and physical intimacy, such as “planning sex”, explicitly discussing needs, using barrier methods, and engaging in non-penetrative physical intimacy.

## Physical Intimacy and Autism

The issue of sensory processing differences identified by Barnett and Maticka-Tyndale (2015) is an important issue when considering the importance of touch, taste, smell, and sound in romantic and intimate relationships. For example, when interpreted as a pleasant sensation, touch plays an important role in interpersonal bonding, erotic and sensual experiences (Jonsson et al., 2015). However atypical sensory processing, a key diagnostic feature of ASD, can result in autistic people experiencing sensory stimuli differently to non-autistic people. Hypo- or hyper-sensitivity can affect any sensory modality and may reduce the pleasantness of close physical contact (Robertson & Baron-Cohen, 2017). This could become a barrier for autistic people seeking to establish and maintain intimate relationships as identified by participants in Barnett and Maticka-Tyndale’s study and warrants further investigation.

Physical intimacy is a key aspect of close relationships across the lifespan in both romantic and non-romantic relationships (Field, 2010; Jakubiak & Feeney, 2017), particularly through “social and affective touch” (Cascio, Moore & McGlone, 2019; Morrison, 2016). Gentle, smooth, rhythmic touch on hirsute skin reach the brain through unmyelinated nerve endings (Olausson et al., 2010), is typically perceived (and intended) as pleasant, and is associated with positive affect (Essick et al., 2010) and erotic sensation (Jonsson et al., 2015). This “slow touch system” likely evolved from allogrooming in primates. Allogrooming plays a key role in encouraging social cohesion, reproductive fitness, and establishing social standing (Dunbar, 2010; Jablonski, 2021). While this outlines the bottom-up aspects of social touch, the top-down aspects include contextual factors and the relationship between the parties involved, which can affect how touch is received and interpreted (Cascio et al., 2019). Affective touch that is perceived as affectionate from an intimate other may result in cognitive-relational changes, such as perceived security, social inclusion and expectations of support if needed; as well as neurobiological changes resulting in reduced stress and positive affect via increase in oxytocin and endogenous opioids and reduced heart rate (Jakubiak & Feeney, 2017). Additionally, slow touch can be perceived as erotic if delivered with the right intensity and speed (Jonsson et al. 2015). Slow touch is typically perceived as arousing on the core erogenous zones of the body, such as the breast and nipples, buttocks, anus and inner thigh, during both masturbation and partnered sexual intimacy, and slow touch can be perceived as erotic anywhere on the body during partnered sexual intercourse (Maister et al., 2020; Nummenmaa et al., 2016). But autistic people may have different experiences.

Through the course of increased research on the clinical features and characterisation of ASD, it has been increasingly recognised that sensory processing differences are a key diagnostic feature. The processing of tactile sensations in ASD shows evidence for typical, hypo- and hyper-sensitivity in the literature, which likely reflects the non-unitary aetiology of the condition (Robertson & Baron-Cohen, 2017). Autistic people experience a higher level of atypical sensory sensations compared to the normative population (Lane et al., 2014; Tomchek & Dunn, 2007). Sensory processing differences can include any and/or all the sensory domains of vision, hearing, smell, taste and touch, as well as vestibular and proprioceptive functions (Lane, Young, Baker, & Angley, 2010); there may also be different phenotypic clusters of sensory processing types which are associated with adaptive functioning and other traits such as inattentiveness and hyperactivity (Scheerer et al., 2022).

Potential behaviours arising from hyper- or hypo-sensitivity range from avoidance to seeking behaviours, hyper-focus, and a range of coping strategies which seek to reduce the impact of sensory processing atypicality (Jones et al., 2003; Robertson & Simmons, 2015). Thus, when autistic people *do* experience atypical sensory processing of touch and tactile sensations, it may affect their perception of pleasantness and eroticism of touch, which in turn may influence the development and maintenance of their close/romantic relationships, as well as their sexual response. For example, while non-autistic people may find gentle, rhythmic touch from others to be pleasant, stress-reducing and even erotic, this may not necessarily be the case for all autistic people.

Management of atypical sensory processes within intimate relationships can be a particular challenge for some autistic people. When personal sensory needs conflict with the preferences of a partner, physical intimacy can be upsetting or painful, with some opting to avoid sexual intimacy altogether (Aston, 2012; Barnett & Maticka-Tyndale, 2015). Given that sexual intimacy is important within relationships and is predicated on an interaction between various physiological, cognitive and emotional processes, it is feasible that autistic people may have some difficulties or differences in their experiences of sexuality and romantic relationships, which is the focus of the current study.

### The Current Study

The current paper extends our previously published research on enablers and barriers of emotional intimacy in romantic relationships, for autistic and non-autistic people, to investigate the role of physical intimacy in intimate relationships. Specifically, the aim was to explore what meaning is attributed to physical intimacy in romantic relationships, what role it plays, and whether there were differences between autistic and non-autistic people in these domains. This is intended to provide a qualitative extension of the quantitative research literature which has established some differences between autistic and non-autistic people on a range of outcomes related to sexuality and relationships. However, this research also seeks to identify common ground amongst the groups. The themes relating to physical intimacy, the meanings associated with it, and the points of convergence and divergence between the two groups are outlined.

## Method

### Participants

Participants (n = 57) included two groups: autistic (n = 31; mean age 32.29 years (SD = 9.07) and non-autistic (n = 26; mean age 33.1 years (SD = 11.51) individuals as reported previously (Sala et al., 2020a, b). All autistic individuals reported a formal diagnosis. Demographic information was collected to characterise the sample, such as age, gender, assigned sex at birth, sexual orientation, educational attainment, religion, and employment; full details are provided in Sala et al. (2020a, b). Throughout we use labels to describe gender identification and sexual orientation when providing examples of participants’ comments. These terms are those they ascribed to themselves, and if interpreted simply, these terms may appear to indicate some contradiction to their statement.

### Materials

Participants in both groups completed an online survey (see Appendix). To screen for autism related traits, the 50-item Autism Spectrum Quotient was used (AQ; Baron-Cohen et al., 2001). The AQ is a self-report screening tool for individuals 16 years and over. Items are rated on a 4-point Likert scale from 1 (definitely agree) to 4 (definitely disagree), then scores of 0 and 1 are coded as 0 with scores of 2 and 3 coded as 1, resulting in a total score (0–50). Higher scores indicate more autism characteristics. A cut-off of ≥ 32 is recommended by the authors of the AQ, identifying 80% of those diagnosed with autism at a 2% false positive rate, (sensitivity = 0.95, specificity = 0.52).

The survey also included an open-ended qualitative questionnaire on experiences of romantic and sexual intimacy designed for this study. Participants were first asked the following question: “Have you ever been in a romantic relationship/s lasting at least one month?”, to which they answered either, “yes, currently”; “yes, in the past”, or “no, never”. Participants were then presented a series of questions about their perspectives on, and experiences of, emotional and physical intimacy in romantic relationships, depending on their experience or lack thereof. Examples of questions are “What does physical intimacy mean for you?”, “Are you comfortable and satisfied with the amount and type of physical intimacy in your romantic relationships?”, “If so, what helps you feel this way?”, “If not, what are the barriers to feeling comfortable and satisfied with the physical intimacy in your relationship?” (For a full list of questions please see Sala et al., 2020a, b).

### Procedure

Prior to data collection, ethical approval was obtained from the overseeing university (DUHREC 2017 − 354), consistent with the Declaration of Helsinki and the National Statement on Ethical Conduct in Human research outlined by the National Health and Medical Research Council of the Government of Australia. Participants were recruited by advertising on social media, via social connection, and through international support groups for autistic individuals and their allies. Individuals were invited to participate in an online survey, then presented with a plain language statement describing the study and indicated their consent by selecting “accept”. Demographic information were collected first; those identifying as autistic reported details regarding their ASD diagnosis, and all participants were asked about other diagnosed mental or physical health conditions. Following this, all participants were directed to the open-ended survey before completing the AQ.

### Qualitative Analysis

As outlined in Sala et al. (2020a, b), an online survey was selected for data collection as this mode of communication has been positively promoted by autistic self-advocates and other researchers because it removes the complexity of navigating non-verbal communication (Benford & Standen, 2009; Davidson, 2008). Data analysis followed the procedure for thematic analysis outlined by Braun and Clarke (2006): reading the data repetitively to familiarise; generating initial codes; grouping codes into themes; reviewing themes; defining and naming themes; and producing the report. Data were imported into NVivo 12 software package to facilitate analysis, and during initial stages, 15% of the autistic participants’ data were randomly selected and read by a colleague familiar with phenomenological methods in order to discuss and verify the themes emerging in initial coding. During thematic definition, 15% of data were given to a separate blind reviewer for thematic coding to verify the interpretations made by the first author. The rate of agreement between reviewers was 93%, with all disagreements resolved.

While knowledge of prior literature on autism and sexuality aided interpretation, data were coded at the descriptive level (Willig, 2012), generating themes which closely reflected the data, focusing on what was stated explicitly by participants in their written responses, drawing on semantic meaning rather than applying theoretical frameworks to interpret underlying meanings. Therefore, an inductive rather than deductive approach was used (Boyzantis, 1998; Willig, 2012). Autistic and non-autistic participants’ data were coded separately; points of similarity and difference between the groups were addressed in later stages of analysis.

## Results

### Demographic Comparisons

As reported in Sala et al. (2020a, b), odds ratio and chi-square analysis were used to compare demographic information of the ASD and N-A participants. Although not all of these differences were statistically significant, autistic participants were more likely to be non-binary gender, non-heterosexual, in non-monogamous relationships, have no prior relationship experience, and be currently unemployed and not studying compared to control participants.

### Physical Intimacy

Three themes emerged in relationship to physical intimacy; these were comfort and bonding, love and sex are different, and sensory sensitivity. Similarities and differences between autistic and non-autistic participants within each theme are summarised in Table 1 and discussed below.

**Table 1:** Brief overview of themes within physical intimacy, similarities and differences between groups

Theme	Similarities	Differences
**Physical Intimacy**		
Comfort and bonding	Physical intimacy as symbol/extension of emotional intimacy	**ASD**: some asexuality, variability in responses, focus on non-sexual contact **N-A**: no asexuality, greater focus on sex being a bonding experience
Love and sex are different	Idea that sex is not always a symbol of love, non-monogamous relationships	**ASD**: some asexuality, variability in responses it terms of why sex may be less exclusive or important **N-A**: mismatched libido, no asexuality
Sensory sensitivity	**ASD only**: sensory needs can make physical affection undesirable or challenging	

### Comfort and Bonding

Physical intimacy was described as important by most participants across both groups (70% of autistic participants, and 100% of non-autistic participants [*z* = 3.05, *p* = .001]). Most participants’ responses indicated that without some form of physical intimacy, their romantic relationships may struggle, or may feel too similar to a friendship. However, not all participants rated sexual intercourse as the most important type of physical affection. Rather, many participants emphasised physical intimacy that reinforces a sense of attachment, such as “hugs or cuddling”, or other tangible connections that “feels comforting and safe and connected” (36, female, heterosexual, N-A).

Some participants highlighted sex was an important peak experience to solidify the bonding within the relationship, a “physical expression of love and belonging” (57, female, bisexual, N-A), and makes them feel “desired and wanted” by their partner (24, male, heterosexual, N-A). However most described regular physical affection (that was less overtly sexual) as being those physical acts that provided them with the greatest sense of reassurance, “skin to skin contact helps me build trust and emotionally bond” (27, male, heterosexual, ASD). This included cuddling, touch, physical proximity, sitting together, and sleeping in the same bed. Some of these acts were described as exclusive and not shared with others, which “makes it particularly special” (36, female, heterosexual, N-A).

It adversely affects my mood and my feelings about my relationship if I don’t get to sleep next to my partner for a prolonged period of time. Ultimately humans are animals and we relate to one another physically. (39, male, heterosexual, N-A).

Some participants also described physical affection as part of intimacy in their non-romantic relationships, “I enjoy physical intimacy with anyone I feel comfortable sharing the experience with and it does not necessarily feel romantic to me…” (30, female, asexual, ASD). Hugs, holding hands and physical proximity were also referenced as forms of bonding learnt within the family context and sometimes shared with friends or pets, which creates intimacy with no sexual potency attached to it.

If I want comfort from him in that way and he says yes then it’s nice, and if he’s not feeling it then I’ll cuddle my pet or engage in one of my interests or something as a way of self-soothing. (24, female, bisexual, ASD).

***ASD.*** While many autistic participants were interested in and enjoyed engaging in sexual acts with lovers or partners, there were several people in the autistic group who identified as asexual, while nobody in the non-autistic group identified this way. This may contribute to the slightly greater variability in what autistic participants found comforting or enjoyable, and tendency to focus on acts such as cuddling and non-sexual physical contact as a form of intimacy and comfort. Not all autistic participants felt that physical intimacy was an important part of their romantic relationships.

We live in a long-distance relationship, which also means that we see each other rarely. So [physical intimacy] can’t play a big role in the relationship to begin with and I don’t mind that. My partner does [mind], and wants way more physical intimacy. (27, agender AFAB, demisexual, ASD).

***N-A.*** Amongst the non-autistic group, none of the participants identified as asexual, and sexual acts were generally referenced as part of their romantic relationships. A stronger sense of sex providing comfort or acting as a “bonding kind of activity” (29, male, heterosexual N-A) came through in the N-A data, though N-A participants also emphasized non-sexual touch and proximity. Taken together, all of the non-autistic participants felt that physical intimacy is an important part of romantic relationships; whereas physical intimacy was less important to autistic participants.

I often feel the most intimate with people I am physically intimate with, I show my trust and vulnerability in sexual ways. Having connected sex builds intimacy for me in a way that is quite different from friendships. (27, female, queer, N-A).

### Love and Sex are Different

Sex was described by some participants in both groups as a symbol of connection and bonding, with some going so far as to say they need a “strong emotional bond to a potential sexual partner” (27, agender, demisexual, ASD) to feel sexual desire, and emphasized the exclusivity of sexual contact with their partner. However, another subset of participants emphasized that sexual desire can exist outside of monogamous romantic relationships, and that sex can be separate to love.

The notion of romance feels dishonest to me … pretending I’m in love with every girl I’m attracted to is an insult to their intelligence … if I ever had the chance, I would be hypersexual (27, male, heterosexual, ASD).

Some participants in both groups talked about having mismatched libidos or different views on the importance of sex compared to their partners. Some described difficulties related to this, and a desire to engage in sexual intercourse with people outside of the relationship. Some participants in both groups also had explicitly consensual non-monogamous romantic relationships, wherein they had committed romantic partnerships with people they loved and usually lived with, but engaged in casual sex with people outside of the relationship.

I wasn’t previously [sexually satisfied] … so I started seeing other people more regularly rather than put that resentment/dissatisfaction on my partner too much. I have a very high sex drive … (28, femme, bisexual, N-A).

In my current relationships my partner and I have the option to sleep with other people from time to time, and it seems to help, although it can obviously be complicated (39, male, heterosexual, N-A)

The desire and drive for sexual and physical intimacy that is separate to loving, ongoing affiliative relationships may be stronger in some people than others.

[I] would like more sex but not necessarily with my partner, who I love and never want to be without … (33, non-binary, heterosexual, ASD).

**ASD.** There was diversity in responses amongst the autistic group. As some participants in this group were asexual and/or experienced gender dysphoria, there were notions of love and romance that were considered separate to sex, and some participants expressed very little or no interest in having sex even if they would like a partner in future, “I could never satisfy a relationship that requires sex or excessive touching” (25, genderflux demi-male, unknown sexual orientation, ASD). For the participants who were not asexual, there were still some who expressed that sex is of lesser importance in their relationships for various reasons, “currently, non-sexual physical intimacy is important with both my wife and girlfriend, mostly because I’m too disable[d] to [have] sex” (38, queer male, ASD). In contrast again, there were some autistic participants who expressed interest in sex, not necessarily within the context of a romantic relationship.

**N-A.** Amongst the non-autistic group, some participants were in non-monogamous relationships as described above, wherein having their sexual needs being met by people outside their loving committed relationships was acceptable and was treated differently to their primary intimate relationship. These participants did not tend to equate this kind of sexual intimacy as being representative of love or commitment. Some participants also indicated that sexual contact is not the most important part of their relationships, and sex itself doesn’t define whether or not a relationship is romantic/intimate. Mismatches in the desired amount of sexual intimacy was also common, and most participants described this as something they navigate.

I’ve gotten to the point in my relationship where [sex] not the main issue, it’s just kind of a nice addition to the relationship (20, female, demisexual, N-A).

### Sensory Sensitivity

This theme was specific to autistic participants, many of whom discussed their experiences of sensory overload or hyposensitivity in relation to physical intimacy. It is important to note that not all autistic participants described sensory processing issues in relation to physical intimacy, therefore it must not be assumed that this phenomenon affects all autistic people. For the participants who described sensory issues, many discussed feeling overwhelmed by too much physical touch, even having “an aversion to touch” (25, genderflux demi-male, unknown sexual orientation, ASD). Some participants described how they can tolerate a certain amount or type of touch, but “a lot of cuddling can startle or tickle” (26, female, heterosexual, ASD), as well as feeling “touched out” over the course of the day, which can reduce the desire to engage in physical intimacy with a partner. For participants whose partners were also autistic, there were descriptions of how each person’s sensory needs needed to factor into the relationship.

[It] took me ages to be okay with even touching other people, but now I’m fine with touching my boyfriend but not really anyone else. I prefer touching my cheek to his instead of kissing, he likes that too … (24, female, bisexual, ASD).

I do not like to be touched, so just cuddling is pretty intimate in our relationship … if physical touch is initiated by surprise, I can get annoyed … (29, female, bisexual, ASD).

Some participants reflected on the challenges that can arise as a result of hypo- or hyper-sensitivity to touch. Current or previous challenges in communicating and navigating their needs around physical touch with partners and others was described as a barrier for seeking future relationships or engaging in sexual acts.

Telling [a sexual partner] they need to use a tool/toy/vibrator if they want me to have an orgasm because I am hyposensitive to touch… seems to always turn into them trying to prove me wrong … (30, female, asexual, ASD).

I thoroughly enjoy kissing and cuddling and very intense foreplay … with a trusted partner, but am not all that interested in penetrative sex … barriers to comfort are sensory overload and my partner’s potential feelings of rejection if I need to take a break [during sex] … (44, “primarily male”, pansexual/queer, ASD).

There were some physically and psychologically adverse experiences outlined by some participants, such as the impact of gender dysphoria, pain, and negative healthcare experiences, on reduced desire for physical intimacy.

I have some physical issues that I’m trying to work out and get diagnosed. Because I have pain often during sex, unfortunately we mostly only do [other sexual practices] for example, rather than [non-penetrative sex]… (26, female, heterosexual, ASD).

## Discussion

The current paper was part of a project aiming to identify enablers and barriers of emotional and physical intimacy for autistic and non-autistic people in romantic relationships. This paper focused on the themes relating to physical intimacy, its role and meaning in romantic relationships. Overall, most participants across the two groups had current or previous relationship experience, and if not, were interested in future relationships. Additionally, most participants in the two groups felt that some form of physical intimacy is important in romantic relationships, with many indicating sexual intimacy is important, though the emphasis on this varied. Some autistic participants described sensory hypo- or hyper-sensitivity and how this impacted their experiences, however this was not a feature for all.

A strong theme across both groups was the notion that physical affection creates a sense of “comfort and bonding” between partners. Both groups described acts such as cuddling, touching and physical proximity make them feel safe and reassured in their romantic relationships. This is consistent with the literature supporting the importance of affective social touch in co-regulation, communication, and positive affect within relationships across the lifespan (Morrison, 2016), and evidence that gentle stroking, hand-holding, hugging and other non-sexual physical acts relate to interpersonal attachment and affective co-regulation processes in many close relationships (Bowlby, 1969; Debrot et al., 2021). Some participants described sexual intimacy as a peak bonding activity between themselves and their partners, though not all participants emphasized this. The importance of sex as an “expression of love” was also found by Meston and Buss (2007) who also found that ‘love’ was rated below “pure attraction to the person” and “experiencing physical pleasure”, suggesting that sexual drive and desire are perhaps more common motivators for sexual acts. Some of our participants expressed similar motivations. While these practices may not be exclusive to romantic partners, some form of physical intimacy, not necessarily sex, was rated as important by most participants and was felt to be an important symbol of attachment within the relationship.

The second theme reflected that sexual desire can exist separately to, and outside of, loving, committed relationships, with some participants across both groups distinguishing sex from love. Across the two groups, some participants were also in consensual non-monogamous relationships. A recent study (Haupert et al., 2017) using a representative adult sample from the United States showed that approximately one fifth of people had experienced a consensual non-monogamous relationship in their lifetime, and this was not confounded by age, education level, religion or a range of other demographic characteristics; however it did covary with gender and sexual orientation. Men and non-heterosexual people (compared to women and heterosexual people) were more likely to have engaged in consensual non-monogamous relationships in the past. The emergence of this theme suggests that, like other human traits such as personality and sex-drive, preferences surrounding monogamy may exist on a spectrum, and/or be influenced by societal norms and expectations. While there seemed to be convergence on the importance of affectionate physical touch in romantic relationships, there was less consensus around the role and symbolic status of sex, both between groups and within the autistic group. Interestingly, some autistic participants in this sample identified as asexual yet still expressed some interest in romantic relationships in future. This finding is consistent with the literature (e.g., Van Houdenhove et al., 2015; Sherrer, 2010), wherein many asexual people still experience romantic, non-sexual attraction towards others and are interested in having dyadic relationships characterised by mutual support, companionship, affection and commitment. Thus, asexuality does not necessarily preclude people from being interested in romantic relationships.

While the autistic and non-autistic participants showed broad similarities across the two themes Comfort and Bonding and Love and Sex are Different, there were some differences in the nuanced experiences therein. There was more variation amongst the autistic participants, as some identified as asexual, and had diverse gender and sexual orientations, and these would be expected to naturally influence their experiences and responses. The autistic participants had a reduced tendency to describe sex as bonding activity, while many non-autistic participants described sex as being pivotal in symbolising love and shared vulnerability. The autistic participants also placed less emphasis on the importance of physical contact in general within their relationships, while all non-autistic participants felt this was important. This leads to the final theme which was specific to autistic participants: that of “sensory sensitivity”.

While not all autistic participants described sensory sensitivity in their responses, many felt overwhelmed or averse to touch, and therefore had to find ways of compensating, tolerating or habituating to physical intimacy with their partners. This is consistent with other qualitative findings about how autistic people use coping and compensatory strategies to manage their sensory needs (Barnett & Maticka-Tyndale, 2015; Robertson & Simmons, 2015). Having a sense of clear communication and control over sensory experiences appeared to help participants feel comfortable with physical intimacy. But if that communication was, or was anticipated to be, not well received, physical touch was considered a barrier to enjoyment and future pursual of relationships. Interestingly, most of the participants in this study who articulated sensory overwhelm were natal females, which may be an artefact of the sample; however it is worth noting. Being able to identify, articulate and manage sensory needs in romantic relationships and sexual experiences appears to be pivotal in supporting the healthy sexual development of autistic people. Given that affective social touch is considered a primary aspect of interpersonal relationships across the lifespan, the fact that some autistic people have atypical tactile sensory processing cannot be ignored.

### Limitations and Future Directions

One major limitation of this study is that although the online survey format was helpful for minimising non-verbal communication and recruiting from a diverse participant pool, it was not possible to ask follow-up questions which may have added to the richness of the data and clarified details relating to participants’ responses. For example, it would have been helpful to ask the autistic participants some follow up questions about their sensory experiences, to understand more clearly how they negotiate their needs. It would have also been interesting to ask participants who had non-monogamous relationship structures how their experience of physical intimacy may or may not be different with various partners, and how they negotiate having an open relationship. Additional limitations of this study include the predominantly female or feminine-identifying participants, despite multiple attempts to recruit more male participants, which may limit the relevance of these themes to males. The participants of this study were also self-selected, which may represent those who are more interested in such topics and may have greater self-awareness and interest in romantic and sexual relationships and have lower support needs.

Future research in sexuality and autism must include consideration of the sensory processing differences which often exist for autistic people. Further research could explore possible links between tactile hypo- or hyper-sensitivity and other factors within relationship-functioning, as well as its possible links with attachment, bonding and erotic sensation. For example, there is some evidence that massage therapies may increase social skills, reduce anxiety, increase tolerance to touch and have positive impacts on attachment bonding and emotion regulation in autistic children (Silva et al., 2011; Walaszek et al., 2018). Therefore, sensitivity to touch in autistic adults might also be remediated by massage therapy or other partnered touch-based interventions, and further research would be paramount in identifying suitability and outcomes for these kinds of intervention and their implications for intimate relationships across the lifespan. The roles of sensory processing differences in other senses should also be explored.

### Clinical Implications

Clinicians providing support in this area, such as psychologists, social workers, sexologists, counsellors, general practitioners, and other mental health and helping professions, would benefit from upskilling in topics related to attachment in intimate relationships, and lifespan perspectives of psychosexual development. In addition, they would benefit from having a cross-disciplinary framework to guide collaboration toward creating a biopsychosocial formulation of difficulties related to relationships and/or sexuality. Such collaboration would assist with identifying areas for intervention from a holistic perspective. For this particular population, autistic adolescents and adults should be supported to access comprehensive education on sexuality and relationships if they are expressing interest and requesting assistance in this area, as the literature suggests they tend to receive inadequate education compared with non-autistic counterparts (Hancock et al., 2017). In doing so, clinicians should be cognisant of the higher prevalence of diversity in gender and sexuality in this population when delivering support and information, and when appropriate, provide linkages with sexual health services, LGBTQIA + resources and helplines, support groups, and other places where people can connect with individuals who have similar experiences and needs. Increased attention should be paid to effective communication, healthy relationships, consent and signs of unhealthy/abusive behaviours. Where possible, autistic adults and adolescents who are having relationship and/or sexual difficulties may benefit from having their partners present in therapeutic intervention sessions to build understanding and foster greater communication, as explicit communication has been identified as helpful within autistic samples in building intimacy (Sala et al., 2020a, b).

## Footnotes

1 Much of this paper concerns matters involving LGBTQIA + issues. At times, definitions surrounding these may be unfamiliar to some readers. To assist, we suggest consulting the following resource of the National LGBTQIA + Health Education Center: https://www.lgbtqiahealtheducation.org/wp-content/uploads/2020/02/Glossary-2022.02.22-1.pdf.
